# Association between the atherogenic index of plasma and hearing loss based on a nationwide cross-sectional study

**DOI:** 10.1186/s12944-024-02119-8

**Published:** 2024-04-29

**Authors:** Zhiyuan Wu, Shu Wang, Xiaowu Huang, Mengyao Xie, Zhijin Han, Chen Li, Shuyi Wang, Qi Tang, Hua Yang

**Affiliations:** 1 Eight-Year Medical Doctor Program, Chinese Academy of Medical Sciences & Peking Union Medical College, Beijing, 100730 P. R. China; 2 Department of Otolaryngology, Peking Union Medical College Hospital, Chinese Academy of Medical Sciences & Peking Union Medical College, No.1 Shuaifuyuan, Dongcheng District, Beijing, 100730 P. R. China; 3 Department of Otolaryngology, Shenzhen Hospital, Southern Medical University, Shenzhen, 518100 P. R. China

**Keywords:** Hearing loss, Atherogenic index of plasma, National Health and Nutrition Examination Survey, Cross-sectional study

## Abstract

**Background:**

Hearing loss (HL) is a worldwide public health issue for which the role of dyslipidemia has not been fully elucidated. This study aimed to use the atherogenic index of plasma (AIP), a well-established serum lipid marker, to investigate the association of dyslipidemia with HL among the general population.

**Methods:**

Participants (*n =* 3267) from the National Health and Nutrition Examination Survey database (2005–2012, 2015–2018) were included in the present study. The AIP was calculated based on the following formula: log10 (triglycerides/high-density lipoprotein cholesterol). HL was defined as a pure-tone average of at least 20 dBHL in the better ear. Weighted multivariable logistic regression, subgroup analysis, generalized additive model, and threshold analysis were adopted to reveal the association between the AIP and HL.

**Results:**

In this study of US adults, a positive association was found between the AIP and high-frequency HL. However, the association between the AIP and low-frequency HL was not as strong. In addition, a reverse L-shaped curve with an inflection point located at -0.27 was detected between the AIP and high-frequency HL, followed by a significant positive association after the inflection point.

**Conclusions:**

The potential of the AIP as a bioindicator for high-frequency HL is noteworthy, and maintaining an AIP value below a certain threshold might provide beneficial outcomes in the management of high-frequency HL.

## Introduction

Hearing loss (HL) is a global public health issue that affects 430 million people [1]. Untreated HL can have detrimental effects on various dimensions of an individual's life. The psychological consequences associated with HL include depressive symptoms, social isolation, and feelings of loneliness [2, 3]. According to a previous study, HL imposes an enormous economic burden, surpassing a monetary value of US$750 billion when considering the annual financial implications [4]. Therefore, the impact of HL is widely acknowledged by various stakeholders, including researchers, clinicians, policy-makers, and individuals suffering from the condition [5].

In everyday life, however, screening for HL is not common and is frequently overlooked due to its considerable financial implications and the perception among patients that it is unnecessary [6]. Consequently, a significant portion of individuals encounter challenges in accessing efficacious and prompt medical interventions. To solve this problem, considerable focus has been directed toward investigating the variables that increase susceptibility to HL, along with the formulation of prompt and efficacious preventive strategies.

The association between hyperlipidemia and HL has been investigated in a variety of studies, including cross-sectional, longitudinal, and interventional studies [7–9]. Hyperlipidemia may increase blood viscosity, leading to reduced blood flow to the labyrinth and tissue damage [10]. In 2001, the atherogenic index of plasma (AIP) was proposed by Dobiásová and Frohlich, which is calculated by a logarithmic transformation of the triglycerides (TG) to high-density lipoprotein cholesterol (HDL-C) ratio and has emerged as a novel and improved lipid and atherogenic marker in recent years [11]. The AIP combines the levels of both TG and HDL-C, providing a comprehensive assessment of dyslipidemia compared to solely considering either lipid type [12].

Since the association between dyslipidemia and HL is neither widely recognized nor fully examined, the purposes of the current study were to evaluate the association between the AIP and HL based on a cross-sectional study of the National Health and Nutrition Examination Survey (NHANES) cohort and to offer valuable insights into the clinical implications of the AIP as a potential bioindicator for HL.

## Materials and methods

### Study population

The NHANES is a comprehensive nationwide survey carried out by the National Center for Health Statistics (NCHS) over a two-year cycle [13]. The NHANES investigates a wide range of components, including in-person interviews conducted at participants' residences to gather information on demographics, socioeconomic status, and diet. Additionally, at the Mobile Examination Center (MEC), well-trained medical personnel conduct the health examinations and are responsible for anthropometry and laboratory testing to collect medical data [14–16].

**Fig. 1:**
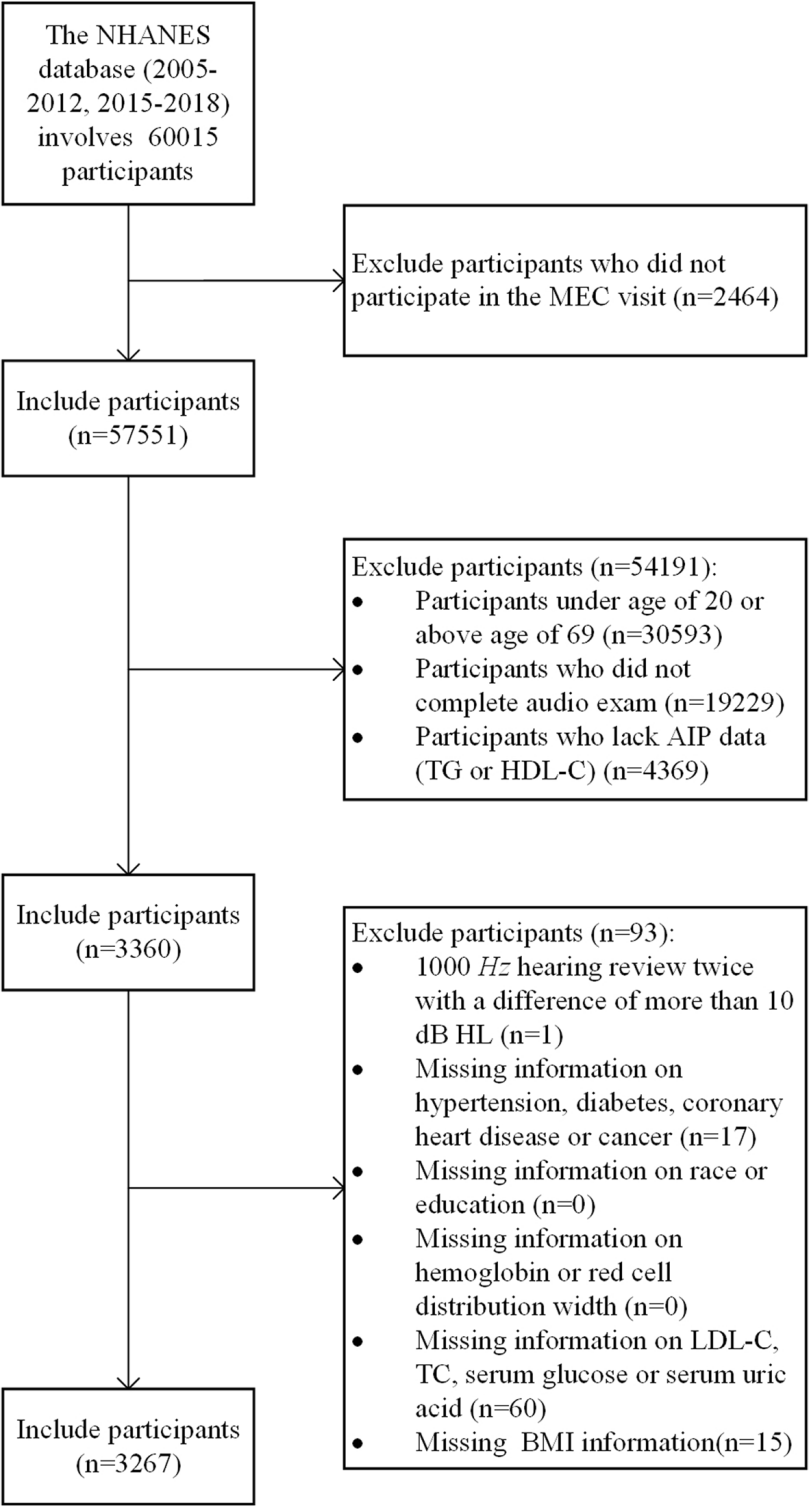
Participant selection flowchart

Data from six NHANES survey cycles (2005–2006, 2007–2008, 2009–2010, 2011–2012, 2015–2016, and 2017–2018) involving 60015 participants were obtained. First, participants who did not participate in the MEC visit were excluded (*n =* 2464). Given that the audiometry exam was limited to individuals aged 20–69 years old, participants falling outside of this specified age range were excluded (*n =* 30593). Subsequently, participants who had incomplete audiometry exam data (*n =* 19229) or TG or HDL-C information (*n =* 4369) were excluded. Meanwhile, a participant with a discrepancy of more than 10 dBHL between the results of the two administered 1000 *Hz* hearing tests was excluded (*n =* 1). Participants lacking complete data on disorders such as diabetes mellitus (DM), hypertension (HTN), coronary heart disease (CHD), and cancer were removed (*n =* 17). Finally, participants with missing information, including race or education (*n =* 0), hemoglobin (Hb) or red cell distribution width (RDW) (*n =* 0), low-density lipoprotein-cholesterol (LDL-C) or total cholesterol (TC) (*n =* 52), serum glucose or serum uric acid (SUA) (*n =* 8), and body mass index (BMI) (*n =* 15), were also removed. The final research sample consisted of 3267 participants (Fig. 1).

### Data collection and definitions

The AIP was considered an exposure variable and was calculated mathematically as log[TG (mmol/L)/HDL-C (mmol/L)] [11]. To acquire a serum sample for lipid profiles, venipuncture was performed using the median cubital, cephalic, or basilic veins in the left arm, unless this arm was unsuitable. Both TG and HDL-C levels were measured using a Roche/Hitachi Cobas 6000 Analyzer Modular P Chemistry Analyzer (Roche Diagnostics, Indianapolis, Indiana, United States).

Following a predetermined protocol provided by the NCHS, trained examiners conducted audiometry in model Delta 142 or model Delta143 sound booths (Acoustic Systems, Austin, Texas, United States) in the MEC. The audiometry procedure included a hearing questionnaire, otoscopic examination, tympanometry, and pure-tone air conduction audiometry. The otoscopic examination was conducted using a Welch-Allyn Model 25020 otoscope (Welch-Allyn, Skaneateles Falls, New York, United States). The tympanometry procedure was performed using the Interacoustics Titan tympanometer (Interacoustic, Assens, Denmark), yielding a range of pressure-compliance values. In the booth, participants were tested with an AD226 model audiometer (Interacoustic, Assens, Denmark) or Audiometric Research Tool (ART) (National Instruments, Austin, Texas, United States), equipped with TDH-49P headphones (Telephonics Corp., Farmingdale, New York, United States) and EARTone 3A model insert earphones (Etymotic Research, Elk Grove Village, Illinois, United States) [17]. The air pure-tone audiometry thresholds for both ears were quantified at seven frequencies (500, 1000, 2000, 3000, 4000, 6000, and 8000 *Hz*). The threshold at a frequency of 1000 *Hz* was assessed twice for each ear.

According to the World Health Organization Guide, a pure-tone average (PTA) of at least 20 dBHL in the better ear was considered an outcome variable for HL in this study [1]. The average thresholds at 500, 1000, and 2000 *Hz* were calculated (the mean value of the 1000 *Hz* threshold was used) for the low-frequency PTA, while the average thresholds at 3000, 4000, 6000, and 8000 *Hz* were calculated for the high-frequency PTA.

### Covariables

Covariables in the study included demographic information and medical data. Therefore, the following covariables were included: age, sex, race, education level, BMI (kg/m^2^), HTN, DM, CHD, cancer, Hb (g/dL), RDW (%), LDL-C (mmol/L), TC (mmol/L), serum glucose (mmol/L), and SUA (μmol/L).

The demographic covariables were all self-reported, including age, sex, race, and education level. Race was classified into four categories: Non-Hispanic White, Non-Hispanic Black, Mexican American, or Other. Education level was classified into three categories, with high school level as the middle category.

BMI was calculated as weight in kilograms divided by height in meters squared (kg/m^2^). Based on the World Health Organization guidelines and the given data, BMI was classified into three groups: normal weight (18.5 kg/m^2^ ≤ BMI < 25 kg/m^2^), overweight (25 kg/m^2^ ≤ BMI < 30 kg/m^2^), and obese (BMI ≥ 30 kg/m^2^) [18].

DM was confirmed if a participant met any of the following standards: (1) self-reported diagnosis, (2) use of antidiabetic medications, (3) random blood glucose levels no less than 11.1 mmol/L, or (4) fasting blood glucose levels no less than 7.0 mmol/L.

CHD was defined as whether participants were ever told by a doctor that they had CHD. Similarly, cancer was defined by whether participants were ever told by a doctor that they had cancer or a malignancy of any kind.

According to the 2017 American Heart Association Blood Pressure Guidelines [19], HTN in this study was defined by one of four standards: (1) systolic blood pressure ≥ 140 mmHg, (2) diastolic blood pressure ≥ 90 mmHg, (3) self-reported HTN, or (4) any use of antihypertensive medication.

### Statistical methods

As recommended by the NHANES Guidelines [20], appropriate weighting techniques were used to address the intricacies of the sample design to ensure that the obtained data were representative at the national level.

AIP tertiles were calculated, and all participants were categorized into 3 groups according to those tertiles: Tertile 1 (-1.12, -0.23), Tertile 2 (-0.23, 0.05), and Tertile 3 (0.05, 1.23). To describe the baseline characteristics of participants, categorical variables are represented by counts and percentages (%), whereas continuous variables are represented by means and standard deviations (SDs). To analyze differences in continuous variables among tertiles, a weighted linear regression model or Kruskal‒Wallis nonparametric analysis of variance was applied to the data, while a weighted chi-square test was used for categorical variables.

Three weighted logistic regression models were developed to examine the relationship between HL and the AIP, namely unadjusted, minimally adjusted, and fully adjusted models, as outlined in the STROBE statement [21]. The unadjusted model (Model 1) involved a univariable logistic regression analysis, while the minimally adjusted model (Model 2) included adjustments for age, sex, race, and education level. The fully adjusted model (Model 3) further expanded on these adjustments by including additional covariables: BMI, HTN, DM, CHD, cancer, Hb, RDW, LDL-C, TC, serum glucose, and SUA. The regression results are presented as odds ratios (ORs) and 95% confidence intervals (CIs).

An interaction test was conducted to identify potential effect modifiers, taking sex, age (categorized as < 40 or ≥ 40 years), race, education level, BMI, HTN, and DM into account. If the observed *P*-value for the interaction was significant, a stratified analysis was conducted.

Additionally, a generalized additive model (GAM) based on smooth curve fitting was employed to explore the nonlinear association between HL and the AIP. When nonlinearity was detected, a threshold effect analysis was performed with the inflection point calculated by a recursive algorithm. The difference between the two-part logistic regression model and the standard logistic regression model was evaluated via the likelihood-ratio test.

Statistical analysis was performed using R (version 4.3.0, http://www.R-project.org) and EmpowerStats (version 5.0.0, www.empowerstats.com). A two-sided of *P* < 0.05 indicated statistical significance in the study.

## Results

### Baseline characteristics

Table 1 illustrates the baseline characteristics of the enrolled participants stratified by AIP tertiles. The study comprised a sample size of 3267 participants, with 1594 individuals identifying as men, accounting for 48.79% of the total sample. In contrast to the participants in Tertile 1 or Tertile 2, the participants in Tertile 3 exhibited certain demographic and health characteristics that differed significantly. Specifically, Tertile 3 consisted of individuals who were older, predominantly male, possessed lower educational attainment, and had greater prevalence of HTN and DM; exhibited elevated Hb, BMI, LDL-C, TC, serum glucose, and SUA; and had lower RDW (*P* < 0.05). Importantly, participants in the higher AIP tertiles demonstrated a greater threshold for high-frequency PTA and a greater ratio of high-frequency HL, with all results achieving statistical significance (*P* < 0.05). However, there was no significant difference between the proportion of patients with low-frequency HL among the different AIP tertile groups.

**Table 1:** Baseline characteristics of the study population

Characteristics	AIP			*P*-value
	Tertile 1 (-1.12, -0.23)	Tertile 2 (-0.23, 0.05)	Tertile 3 (0.05, 1.23)	
Age (years)	42.48 ± 14.90	44.15 ± 14.24	45.69 ± 13.83	< 0.001
Sex, n (%)				< 0.001
Male	384 (35.26)	540 (49.59)	670 (61.52)	
Female	705 (64.74)	549 (50.41)	419 (38.48)	
Race, n (%)				< 0.001
Non-Hispanic White	319 (29.29)	366 (33.61)	419 (38.48)	
Non-Hispanic Black	363 (33.33)	239 (21.95)	149 (13.68)	
Mexican American	110 (10.10)	154 (14.14)	188 (17.26)	
Other Race	297 (27.27)	330 (30.30)	333 (30.58)	
Education level, n (%)				< 0.001
Below high school	172 (15.79)	221 (20.29)	263 (24.15)	
High school	215 (19.74)	242 (22.22)	234 (21.49)	
Above high school	702 (64.46)	626 (57.48)	592 (54.36)	
BMI (kg/m^2^)	27.02 ± 14.90	29.42 ± 14.24	31.22 ± 13.83	< 0.001
Hypertension, n (%)				< 0.001
Yes	341 (31.31)	423 (38.84)	507 (46.46)	
No	748 (68.69)	666 (61.16)	582 (53.44)	
Diabetes, n (%)				< 0.001
Yes	99 (9.09)	184 (16.90)	270 (24.79)	
No	990 (90.91)	905 (83.10)	819 (75.21)	
Coronary heart disease, n (%)				0.317
Yes	18 (1.65)	27 (2.48)	27 (2.48)	
No	1071 (98.35)	1062 (97.25)	1062 (97.25)	
Cancer, n (%)				0.382
Yes	59 (5.42)	49 (4.50)	63 (5.79)	
No	1030 (94.58)	1040 (95.50)	1026 (94.21)	
Hb (g/dL)	13.68 ± 1.45	14.08 ± 1.53	14.51 ± 1.45	< 0.001
RDW (%)	13.42 ± 1.36	13.34 ± 1.37	13.27 ± 1.23	0.015
LDL-C (mmol/L)	2.68 ± 0.79	3.05 ± 0.88	3.16 ± 0.99	< 0.001
Serum glucose (mmol/L)	5.18 ± 1.33	5.58 ± 1.79	6.13 ± 2.38	< 0.001
SUA (μmol/L)	287.50 ± 70.83	321.38 ± 78.52	355.41 ± 87.20	< 0.001
TC (mmol/L)	4.72 ± 0.95	4.93 ± 1.02	5.20 ± 1.10	< 0.001
Low-frequency PTA (dBHL)	8.28 ± 21.74	8.54 ± 8.11	9.74 ± 8.65	< 0.001
High-frequency PTA (dBHL)	16.64 ± 24.63	18.27 ± 14.83	21.91 ± 17.02	< 0.001
Low-frequency HL, n (%)				0.141
Yes	90 (8.26)	102 (9.37)	117 (10.74)	
No	999 (91.74)	987 (90.63)	972 (89.26)	
High-frequency HL, n (%)				< 0.001
Yes	310 (28.47)	382 (35.08)	472 (43.34)	
No	779 (71.53)	707 (64.92)	617 (56.66)	

### Association between the AIP and HL

All models demonstrated positive associations between the AIP and high-frequency HL (Model 1: OR = 2.37, 95% CI: 1.89–2.98; Model 2: OR = 1.44, 95% CI: 1.07–1.95; Model 3: OR = 1.35, 95% CI: 0.96–1.89). Furthermore, the observed results maintained statistical significance even when considering the AIP as a categorical variable in the unadjusted model and in Tertile 3. Specifically, there was a consistent increase in the prevalence of high-frequency HL across AIP tertiles where statistically significant trends existed in all three models, with the highest tertile demonstrating the greatest OR compared to the lowest tertile (*P* for trend < 0.05). The complete results are illustrated in Table 2.

**Table 2:** Results of logistic regression analysis between the AIP and high-frequency HL

Characteristics	Model 1		Model 2		Model 3	
	OR (95% CI)	*P* value	OR (95% CI)	*P* value	OR (95% CI)	*P* value
AIP	2.37 (1.89, 2.98)	< 0.001	1.44 (1.07, 1.95)	0.017	1.35 (0.96, 1.89)	0.085
AIP (Tertile)						
Tertile 1 (-1.12, -0.23)	1-reference		1-reference		1-reference	
Tertile 2 (-0.23, 0.05)	1.36 (1.13 1.63)	< 0.001	1.13 (0.89, 1.43)	0.302	1.13 (0.88, 1.45)	0.350
Tertile 3 (0.05, 1.23)	1.92 (1.61, 2.30)	< 0.001	1.36 (1.07, 1.72)	0.011	1.31 (1.01, 1.71)	0.044
*P* for trend	< 0.001		0.010		0.043	

Model 1: No adjustment for covariables

Model 2: Adjusted for age, sex, race, and education level

Model 3: Adjusted for age, sex, race, education level, BMI, HTN, DM, CHD, Hb, RDW, LDL-C, TC, serum glucose, and SUA

In the unadjusted model, a positive relationship between the AIP and low-frequency HL was observed (Model 1: OR = 1.40, 95% CI: 0.97–2.02). Interestingly, with confounding covariables under consideration, no significant association was found between an increasing AIP and low-frequency HL. In addition, no significant trend was observed after adjustment for covariables. The findings are shown in Table 3.

**Table 3:** Results of logistic regression analysis between the AIP and low-frequency HL

Characteristics	Model 1		Model 2		Model 3	
	OR (95% CI)	*P* value	OR (95% CI)	*P* value	OR (95% CI)	*P* value
AIP	1.40 (0.97, 2.02)	0.069	1.03 (0.69, 1.54)	0.868	0.96 (0.61, 1.50)	0.859
AIP (Tertile)						
Tertile 1 (-1.12, -0.23)	1-reference		1-reference		1-reference	
Tertile 2 (-0.23, 0.05)	1.15 (0.85, 1.54)	0.365	1.04 (0.76, 1.43)	0.810	1.07 (0.76, 1.50)	0.705
Tertile 3 (0.05, 1.23)	1.34 (1.00, 1.78)	0.049	1.07 (0.78, 1.47)	0.657	1.03 (0.72, 1.48)	0.853
*P* for trend	0.048		0.657		0.874	

Model 1: No adjustment for covariables

Model 2: Adjusted for age, sex, race, and education level

Model 3: Adjusted for age, sex, race, education level, BMI, HTN, DM, CHD, Hb, RDW, LDL-C, TC, serum glucose, and SUA

### Subgroup analysis

The association between the AIP and high-frequency HL was further analyzed by a test for interaction, where a significant interaction with the AIP was found for BMI (*P* for interaction* =* 0.017). The AIP was significantly positively associated with high-frequency HL among overweight participants (OR = 2.02, 95% CI: 1.10–3.71) after stratification by BMI. In addition, statistically significant trends were found for participants in the overweight strata (*P* for trend < 0.05) (Table 4).

**Table 4:** Results of subgroup analysis for associations between the AIP and high-frequency HL

Characteristics	OR (95% CI)				*P* for trend	*P* for interaction
	Total	Tertile 1 (-1.12, -0.23)	Tertile 2 (-0.23, 0.05)	Tertile 3 (0.05, 1.23)		
BMI						0.017
Normal weight	0.63 (0.32, 1.26)	1-reference	0.98 (0.62, 1.56)	0.72 (0.41, 1.24)	0.276	
Overweight	**2.02 (1.10, 3.71)**	1-reference	0.90 (0.56, 1.44)	**1.68 (1.05, 2.69)**	0.017	
Obese	1.47 (0.85, 2.55)	1-reference	**1.59 (1.04, 2.42)**	1.52 (0.99, 2.33)	0.104	

Statistically significant ORs and their 95% CIs are shown in bold

Adjusted for age, sex, race, education level, BMI, HTN, DM, CHD, Hb, RDW, LDL-C, TC, serum glucose, and SUA

### Nonlinear relationships

**Fig. 2:**
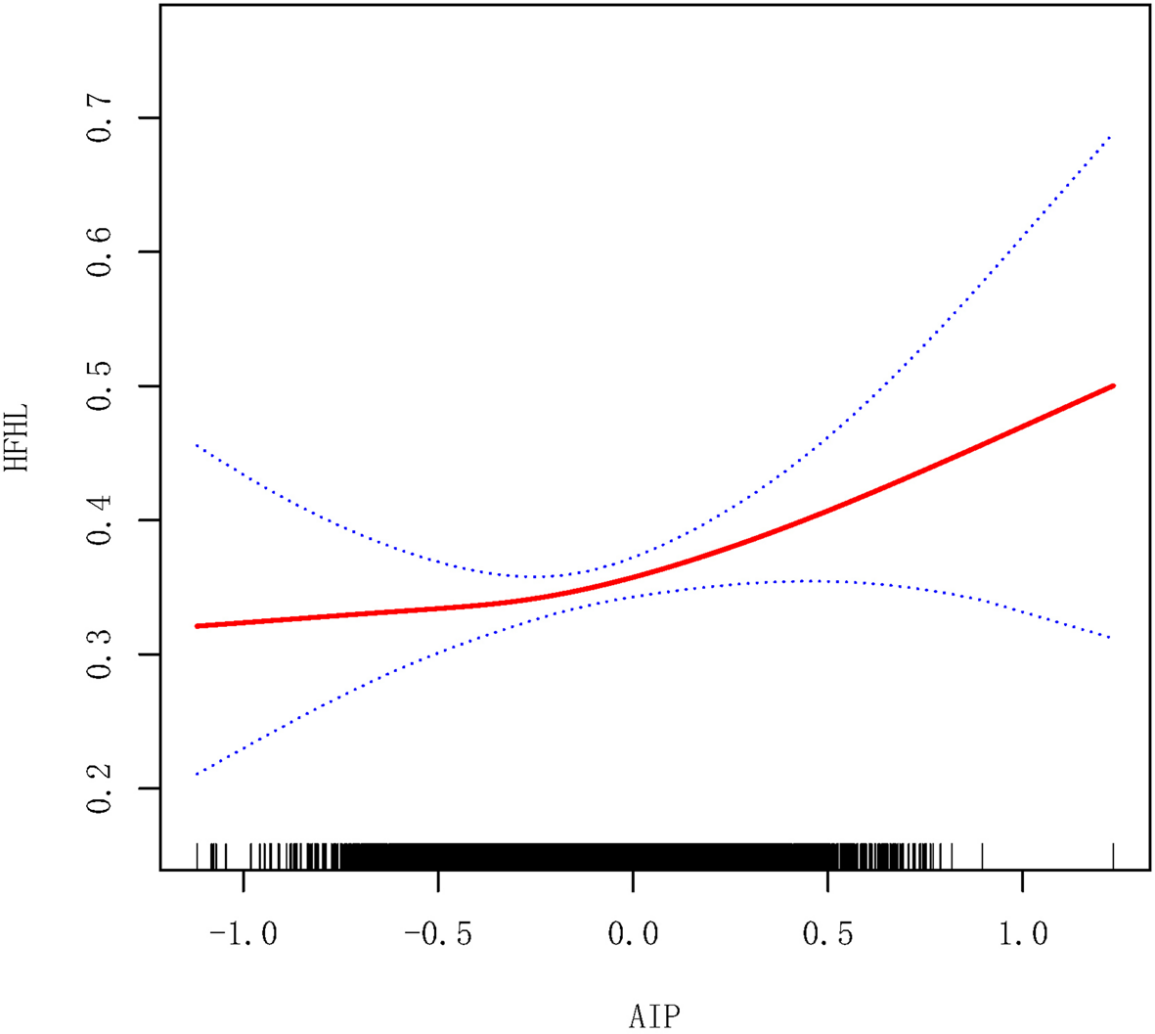
GAM curve of the nonlinear association between the AIP and high-frequency HL. The red solid line represents the probability of high-frequency HL occurrence, and the blue dotted line represents the 95% CI curve

The GAM was employed to validate and reinforce the findings obtained above by detecting the nonlinear relationship between the AIP and high-frequency HL, revealing a reverse L-shaped relationship (Fig. 2). In the following threshold effect analysis, an inflection point of -0.27 was identified. After the inflection point, there was a significant positive association between the AIP and high-frequency HL (OR = 1.83, 95% CI: 1.15–2.91). Nevertheless, no such significant association was found before the inflection point (Table 5).

**Table 5:** Results of the threshold effect analysis for AIP and high-frequency HL

Models	Adjusted OR (95% CI), *P* value
Standard linear model	1.35 (0.96, 1.89), 0.085
Two-part logistic regression model	
Inflection point	-0.27
AIP < -0.27	0.54 (0.20, 1.49), 0.234
AIP > -0.27	1.83 (1.15, 2.91), 0.011
Log likelihood ratio	0.062

Adjusted for age, sex, race, education level, BMI, HTN, DM, CHD, Hb, RDW, LDL-C, TC, serum glucose, and SUA

## Discussion

In this representative sample from the United States, which included 3267 participants, a significant positive association between the AIP and high-frequency HL was found in both the unadjusted and partially adjusted models. In addition, a reverse L-shaped curve was detected for the association between the AIP and high-frequency HL considering nonlinearity, and a significant positive association after the inflection point of -0.27 was also found, suggesting that the AIP has the potential to be a reliable monitoring indicator for underlying high-frequency HL.

The impact of hyperlipidemia on auditory function is intricately linked to the physiological attributes of the circulatory system of the inner ear. The cochlea relies on the vasculature for nutrients and oxygen [22]. From an anatomical perspective, the internal auditory artery is classified as a non-compensatory anastomotic terminal artery [23], supplying blood to both the cochlea and auditory nerve alone. As a result, the cochlea exhibits a heightened susceptibility to any minor fluctuation in blood flow. Regarding the adverse impacts of hyperlipidemia on microcirculation, elevated cholesterol levels inside red blood cells have a direct impact on their ability to deform and transport oxygen, resulting in hindered passage through the microcirculatory system [24]. In instances of hyperlipidemia, the inner ear tissue may experience unsatisfied metabolic demands and be impacted by decreased oxygen availability.

Statins are a widely prescribed category of lipid-lowering medications that act by limiting cholesterol synthesis. Statin use has been reported to impair hearing in vitro, in animal models, and in clinical settings. In murine cochlear nerve cells, simvastatin induces neurodegeneration and apoptosis by blocking the mevalonate pathway [25]. In a zebrafish animal model, simvastatin caused hair cell death [26]. In a clinical cohort of aging individuals, participants taking statins had a lower word recognition rate than those who did not [27]. However, studies have suggested that statins can protect hearing by lowering plasma cholesterol levels (i.e. changing the AIP value) [28–30]. Notably, statins have a cholesterol-independent effect that arises from the pleiotropy of HMG-CoA reductase (3-hydroxy-3-methyl glutaryl coenzyme A reductase), warranting further investigation into the complicated relationship between statin use and hearing [31].

Contrary to the present findings in high-frequency HL, no significant association between increasing AIP and low-frequency HL was found in this study. The base-to-apex high-to-low-tone tonotopic architecture of the basilar membrane of the cochlea allows it to perceive distinct sound frequencies [32]. Specifically, the basal part of the cochlea mainly detects high-frequency tones, while the apical part detects low-frequency tones. Despite the specific frequency, it is essential for sound waves to travel in an upward direction via the basilar membrane. Consequently, the perception of high-frequency tones is associated with a much narrower region of cochlear involvement than that of low-frequency tones, which elicits a broader area of cochlear stimulation. In other words, the basilar membrane responsible for perceiving high-frequency sounds is prone to experiencing tiredness more readily. Previous research has suggested that fluctuations in calcium ion equilibrium throughout the cochlea may partly explain differences in susceptibility [33]. Additionally, the basilar membrane hair cells near the basal labyrinth have fewer antioxidant enzymes than those at the apex, rendering them more sensitive to stress such as free radicals [34]. According to the histology of the cochlear nerve, the outer laminae, including nerve fibers from the basal coil, are mainly responsible for detecting high-frequency auditory stimuli. In contrast, the inner laminae are composed of nerve fibers from the apex that specialize in perceiving low-frequency auditory stimuli [35]. The impact of alterations in the microenvironment, such as ischemia and hypoxia, primarily influences the response of peripheral nerves to high-frequency stimuli.

During the study, it was observed that BMI serves as a significant modifier in the relationship between the AIP and high-frequency HL. A previous study reported that obesity was related to dyslipidemia characterized by increased TC and decreased HDL-C, leading to a greater AIP [36]. Moreover, researchers have shown that oxidative stress induced by a high-fat diet can cause HL [37]. Obesity may contribute to HL via oxidative stress, as noted by prior studies [36, 38], although further investigation to clarify the underlying mechanism is still needed.

With all covariables considered, the relationship between the AIP and high-frequency HL varied on each side of the inflection point. When the AIP was greater than -0.27, it was significantly and positively associated with high-frequency HL. This finding suggests that early therapeutic intervention to manage blood lipid levels is imperative to avoid the potential further development of high-frequency HL.

### Strengths and limitations

One notable strength of this research lies in its substantial sample size, where data from six NHANES cycles were used. Second, the NHANES database has a comprehensive weighted design, allowing it to be a perfect representative of the overall United States population. Second, the NHANES employs standardized protocols for data collection by experienced professionals. All blood samples were tested in a uniform laboratory setting, minimizing potential bias. Additionally, confounding factors that might interact with the AIP were considered, and stratified analysis was performed on covariables with a significant interaction to identify potential special populations. Furthermore, both a GAM and threshold analysis were applied to effectively analyze the underlying nonlinear associations and inflection point.

However, limitations inherent in the current research are unavoidable. First, this study is characterized by its observational nature, which limits the ability to establish causation. Therefore, it is important to approach the current results with caution, and more prospective investigations are required to ascertain the precise association between the AIP and susceptibility to HL. Second, despite efforts to adjust for as many factors as possible, it is vital to acknowledge that there could be unmeasured potential confounders, such as dietary patterns. Finally, it should be noted that this research did not include any data pertaining to the management of dyslipidemia. Hence, it is imperative that further studies be conducted to explore the impact of the combined impact of baseline and divergence AIP scores on the progression of HL.

## Conclusions

The potential of the AIP as a biomarker for high-frequency HL is noteworthy, with a positive association found between the AIP and high-frequency HL. In individuals with an AIP greater than -0.27, a greater AIP was significantly associated with a greater occurrence of high-frequency HL. These findings indicate that controlling the AIP below a certain threshold might provide beneficial outcomes in the prevention and management of high-frequency HL, and it is promising to use the AIP as a bioindicator of high-frequency HL.

## Data Availability

The data in the current study are available at https://wwwn.cdc.gov/nchs/nhanes/Default.aspx.

## Abbreviations

AIP: Atherogenic index of plasma
BMI: Body mass index
CI: Confidence interval
GAM: Generalized additive model
Hb: Hemoglobin
HDL-C: High-density lipoprotein cholesterol
HL: Hearing loss
LDL-C: Low-density lipoprotein cholesterol
MEC: Mobile Examination Center
NCHS: National Center for Health Statistics
NHANES: National Health and Nutrition Examination Survey
OR: Odds ratio
PTA: Pure-tone average
RDW: Red cell distribution width
SUA: Serum uric acid
TC: Total cholesterol
TG: Triglyceride
